# An Unsupervised Smart App–Optimized HIV Self-Testing Program in Montreal, Canada: Cross-Sectional Study

**DOI:** 10.2196/10258

**Published:** 2018-11-27

**Authors:** Nitika Pant Pai, Megan Smallwood, Laurence Desjardins, Alexandre Goyette, Krisztian G Birkas, Anne-Fanny Vassal, Lawrence Joseph, Réjean Thomas

**Affiliations:** 1 Department of Medicine McGill University Montreal, QC Canada; 2 Division of Clinical Epidemiology Research Institute of McGill University Health Centre Montreal, QC Canada; 3 Clinique Médicale L'Actuel Montreal, QC Canada

**Keywords:** feasibility, HIV, impact, mobile phone, MSM, self-testing

## Abstract

**Background:**

Although HIV self-testing strategies have been recommended by the World Health Organization, HIV self-tests are not yet approved in Canada. Currently approved HIV self-tests offer toll-free lines that are insufficient for initiating expedited linkages to counseling and care, accurate interpretation, and support during HIV self-testing. We developed an innovative, multilingual software app called HIVSmart! to plug these gaps.

**Objective:**

This study aimed to test our app-optimized oral HIV self-testing strategy for feasibility in men who have sex with men (MSM) who presented to test at a large sexual health clinic (Clinique Médicale L’Actuel) in Montreal.

**Methods:**

Between July 2016 and February 2017, we offered a strategy consisting of the OraQuick In-Home HIV Test (an investigational device) and a tablet installed with the HIVSmart! app to study participants, who presented at a private office in the clinic, mimicking an unsupervised home environment. We evaluated the strategy for its feasibility, acceptability, and preference. Using the HIVSmart! app, participants were guided through the self-testing process. We determined feasibility with a metric defined as the completion rate, which consisted of the following 3 steps: (1) self-test conduct; (2) self-test interpretation; and (3) linkages to care. Participants independently performed, interpreted, recorded their self-test and result, engaged in pre- and posttest counseling, and sought linkages to care. Laboratory tests (p24, Western Blot, and RNA), as per country algorithms, were expedited, and linkages based on the rapid test status were arranged.

**Results:**

Mean age of the 451 participants enrolled was 34 (range, 18-73) years. Of all participants, 97.1% (438/451) completed and submitted the survey through the HIVSmart! app. In total, 84.7% (371/438) of the participants were well educated (beyond high school) and 52.5% (230/438) had been tested within the past 6 months. Of the 451, 11.5% (52/451) were on pre-exposure prophylaxis. Feasibility (completion rate), an average proportion of the 3 steps, was computed to be 96.6% (419/451). The acceptability of the strategy was high at 98.5% (451/458). A majority of the participants (448/451, 99.3%) were found to be self-tested and lab-confirmed negative and were counseled after self- and rapid tests. In total, 0.7% (3/451) of the participants who self-tested positive and were lab-confirmed positive were linked to a physician within the same day. Furthermore, 98.8% (417/422) of the participants found the app to be useful and 94.0% (424/451) were willing to recommend it to a friend or partner.

**Conclusions:**

The HIVSmart! app-optimized strategy was feasible, accepted, and preferred by an educated, urban MSM population of Montreal. With the app, participants were able to perform, interpret, store results, and get rapidly linked to care. The HIVSmart!-optimized, self-testing strategy could be adapted and contextualized to many at-risk populations within Canada and worldwide, thereby maximizing its public health impact.

## Introduction

In 2014, the Joint United Nations Programme on HIV/AIDS released its 90-90-90 targets, calling for 90% of those living with HIV to be tested, 90% of those tested positive to be on treatment, and 90% on those on treatment to remain virologically suppressed [1]. To reach the first 90 by 2020, it is imperative that we identify those who are living unaware of their HIV status. One such strategy that has the potential to reach the undiagnosed is HIV self-testing. In 2016, the World Health Organization recommended the scale-up of self-testing as an additional alternative to the conventional HIV testing services [2,3]. HIV self-tests are now available for use, sale, and distribution in many countries. More than 59 countries have HIV self-testing policies in place [4-7]. The benefits of self-testing include privacy, confidentiality, convenience, engagement in self-care, empowerment, and proactivity in seeking health [8]. The potential concerns with HIV self-testing are the rapid establishment of linkages for self-testers to counseling and care, rapid reporting of false-negative results in the acute window period, and affordability of self-tests. Nonetheless, benefits of HIV self-testing are generally understood to outweigh the associated risks [8,9].

Evidence and data on self-testing in Canada are sparse [10-12]. Neither oral nor blood-based tests are approved yet by Health Canada [4]. Canadian policies on HIV self-testing are in development. Recently, we surveyed Canadian stakeholders involved in HIV testing initiatives across the country and found that many were in favor of self-testing but were concerned about linkages to care and accuracy of self-tests. Furthermore, HIV self-testing strategies were perceived to pose an economic threat to the prevailing HIV testing models in Canada [13]. While global research on the implementation of HIV self-testing has increased exponentially, few studies have explored self-testing in the Canadian context. To date, a hypothetical self-testing study used focus groups or surveys of attitudes and acceptability, while another evaluated a self-testing strategy in a low-risk student population [11,12]. Thus, implementation research evidence on HIV self-testing is needed for Canada.

In any setting, understanding the context in which self-testing should be implemented is critical to its success. In Canada, the HIV epidemic is disproportionally represented in key populations, such as men who have sex with men (MSM), injection drug users (IDUs), Aboriginal populations, and immigrants from HIV endemic countries. Approximately 18%-25% of Canadian MSM populations are unaware of their HIV-positive status [14], and the number may be proportionally higher for IDUs, Aboriginals, and immigrants, which underscores the need for accessible HIV self-testing services.

## Methods

### HIVSmart!

**Figure 1:**
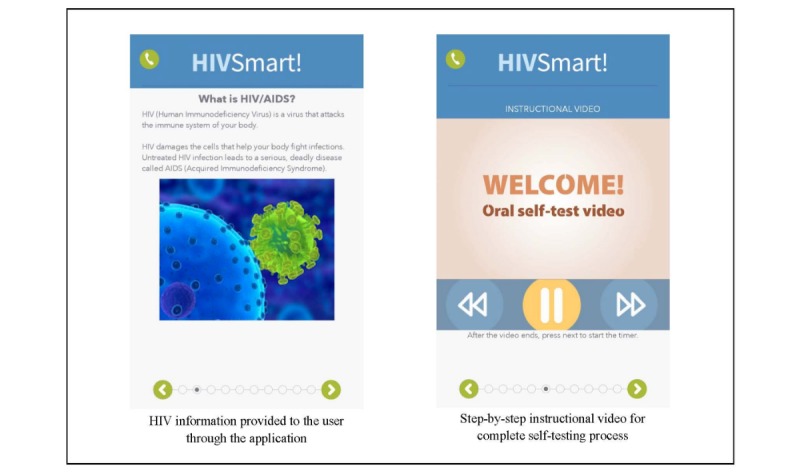
Screenshots of the HIVSmart! app.

HIVSmart! (Figure 1), a Canadian innovation funded by Grand Challenges Canada [15], (which is funded by the Government of Canada), is a multiplatform smartphone-, tablet-, or Web-based (Android, iPhone, and iPad) confidential software app. This study was funded by the Canadian Institutes of Health Research [16]. HIVSmart! plugs gaps in the self-testing process, works with any approved HIV self-test, engages, and proactively informs any intended user of an HIV self-test. It interprets and stores data confidentially, links users to counseling or care within a rapid turnaround time, and encourages them to stay in care. For this study, we adapted the HIVSmart! app-based program for Canadians. Reverse innovation entailed language adaptations (French-Canadian) and customizations to the US Food and Drug Administration-approved oral self-test products and obtaining Health Canada’s Investigational Testing Authorization for research.

HIVSmart! is unique in that it is a complete app-based solution. It is an improvement from the Web prototype of HIVSmart!, which was initially evaluated in health care professionals of South Africa and students of McGill University [12,17]. This novel app-based solution is currently being tested at scale in South Africa [18].

### Study Participants and Design

Between July 2016 and February 2017, we conducted a cross-sectional feasibility study at Clinique Médicale L’Actuel, a private, sexual health clinic specializing in testing and treatment of HIV and sexually transmitted infections, located in urban Montreal. Ethical approvals were obtained from Clinique L’Actuel’s independent review board (Veritas Institutional Review Board), and the McGill University Health Centre Research Ethics Board. Study procedures were duly followed and complied with the regulations stipulated by the Institutional Review Boards of McGill University Health Centre and Veritas.

In high risk, MSM populations presenting to the clinic, we set out to achieve the following objectives. We aimed to evaluate the feasibility, acceptability, and preference for an unsupervised HIV self-testing strategy that involved a self-test (OraQuick In-Home HIV Test) and an accompanying optimized app (HIVSmart!) in an unsupervised setting that mimicked a home environment.

Participants were recruited by convenience sampling; clinic staff recruited participants during routine and drop-in clinic visits. The study was advertised via flyers, social media, and through e-newsletters.

Participants were deemed eligible to join the study if they were 18 years or older, self-identified as an MSM, of unknown HIV status, and comfortable using smartphones or tablets. Participants receiving pre- or postexposure prophylaxis (PrEP or PEP) antiretrovirals for prevention (ARVs) were also invited to participate so as to not exclude these high-risk groups. Participants were excluded if they self-reported a previously confirmed positive HIV diagnosis.

**Figure 2:**
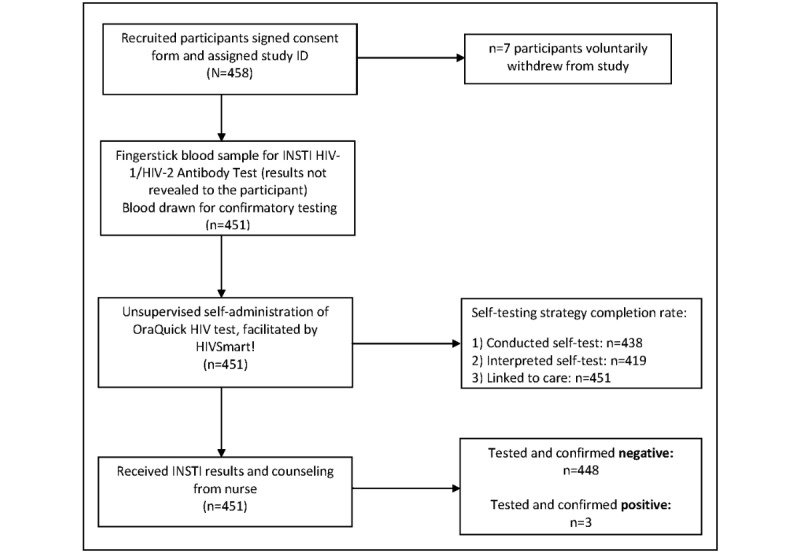
Flow of study participants. ID: identifier.

A flow chart outlining the study flow is represented in Figure 2. Once deemed eligible to participate, the study procedure was explained to participants. Informed consent (written) was obtained from all participants before beginning the study. Participants could withdraw consent at any point throughout the study. At this time, participants were informed about the strategy that involved deidentified data collection on a confidential, compliant secure server.

### Study Procedure

As per Health Canada’s product monographs and Investigational Testing Authorization recommendations, the usual clinic procedures were followed in the study. Following participants’ informed consent and agreement to participate, participants were explained about data storage and confidentiality. Following this, a rapid test (INSTI HIV-1/HIV-2 Antibody Test; bioLytical Laboratories) was performed by the research nurse. However, the rapid test result was not revealed to participants at this time. Blood was drawn for laboratory-based confirmatory testing, following the clinic’s protocol (Centre hospitalier de l’Université de Montréal): p24 antigen testing and anti-HIV-1/2 testing for all samples, confirmed by Western Blot if abnormal. Samples underwent RNA testing in case of suspected acute HIV infection. Lab testing conformed to the national testing algorithms.

Participants were then brought to a private office in the clinic and provided the OraQuick Test kit along with a tablet installed with HIVSmart!. The process of self-testing went thus: participants were left alone (unsupervised) to navigate the app on the tablet, stage their personal risk for HIV, perform the self-test as per instructions on HIVSmart!, and conduct the test unsupervised, mimicking a home environment. All participants followed instructions on pretest counseling, staging, conducting results, and storing their results on screen. Participants were asked to wait for their test result using a timer built into the app. They were encouraged to use the phone number provided in the app to call for counseling or assistance at any time and respond to a research questionnaire within the app. Upon completion of the self-testing process, participants were offered the choice of calling the provided phone number to receive posttest counseling or receiving face-to-face posttest counseling with a research nurse. Phone-based counseling was rapidly followed by face-to-face counseling with the research nurse. Upon meeting with the research nurse, who interpreted and recorded the self-test results in real-time separately, the INSTI rapid tests results were provided to participants and they were offered posttest counseling based on their INSTI results. Participants with positive HIV results were seen by a physician immediately for follow-up care. Participants with negative self-tests were encouraged to return for retesting in 3 months. As per the Institutional Review Board recommendations, all participants were offered a modest compensation for their time (Can $20).

### Outcomes and Metrics

The primary outcome of this study was the feasibility of the HIVSmart! self-testing strategy. Feasibility was documented by the metric completion rate, defined as the number of participants who successfully completed all steps of the self-testing procedure, over the total number of consenting participants. Steps were defined as follows: (1) self-test conduct; (2) self-test interpretation; and (3) seeking linkage to care. It was computed as an average proportion of the completion for each step in the procedure.

Secondary outcomes were patient-centered outcomes (acceptability of the strategy, preference for counseling, and costs) that were collected using the metrics listed below:

1. *Acceptability*: the number of participants who consented to evaluate the strategy, over the total number of people approached for recruitment. Refusals were documented by staff at the clinic.
2. *Preference for (counseling)*: participants were asked to respond to a questionnaire, regarding preferences for suitable posttest counseling options, in a hypothetical scenario where self-tests were available for purchase in pharmacies.
3. *Preference for (cost)*: participants were asked in a questionnaire to select a price point at which self-tests should be made available in Canada (<Can $10, Can $10-20, Can $20-40, and >Can $40).
4. *Usefulness of the app*: participants were asked if the app was found to be useful in assisting them in the self-testing process.
5. *Recommendations to friends or partners*: participants were asked if the app would be recommended by them to their friends or partners.

### Data Analyses and Sample Size Estimations

Our sample size was estimated using the completion rate as the main outcome. Estimating a conservative completion rate of 70% with a CI width of 10% (SD 0.0565%), we calculated a target sample size of 400.

Basic proportions for demographics and outcomes obtained through the HIVSmart! app were computed with their 95% CIs. A stratified analysis comparing participants receiving ARVs (PrEP or PEP) versus no ARVs was conducted for the main feasibility outcome variables with Fisher’s exact test, which determines significant differences between groups (StataCorp, 2013, *Stata Statistical Software: Release 13,* StataCorp LP).

## Results

### Participant Characteristics

A total of 451 participants met the eligibility criteria and accepted to evaluate the HIVSmart! self-testing strategy (Table 1); 97.1% (438/451) of the participants submitted the HIVSmart! survey (Table 2).

**Table 1:** Descriptive characteristics of study participants.

Characteristics		Value (N=451)
Age (years), mean (range)		33.6 (32.6-34.7)
**Antiretrovirals for prevention status, n (%)**		
	None	394 (87.4)
	Postexposure prophylaxis	5 (1.1)
	Pre-exposure prophylaxis	52 (11.5)

Participants were well educated, with 84.7% (371/438) educated beyond high school; 79.5% (348/438) were employed, and 52.5% (230/438) had been tested in the past 6 months. Participants self-identified themselves as male (98.6%, 432/438) and homosexual (91.3%, 400/438); 96.1% (421/438) of participants were sexually active, with 25.1% (110/438) stating 6-10 partners in the past 6 months; 21.9% (96/438) stating ≥11; and 70.1% (307/438) engaged in condom-less sex. In total, 11.5% (52/451) participants were currently taking PrEP and 1.1% (5/451) were taking PEP.

Acceptability was high at 98.5% (451/458); 7 participants refused to participate or withdrew themselves from the study.

Feasibility, as documented by the completion rate of the HIVSmart! self-testing strategy, was computed by taking an average of the 3 steps of the self-testing strategy—(1) self-test conduct; (2) self-test interpretation; and (3) linkages to care.

1. *Self-test conduct:* 97.1% (438/451) of the participants conducted the self-test successfully.
2. *Self-test interpretation*: 92.9% (419/451) of participants interpreted their self-test successfully.
3. *Linkages to care*: 100% (451/451) of the participants sought linkages to care successfully.

To compute feasibility, we took an average proportion of the 3 steps highlighted above, resulting in the overall feasibility of 96.7%.

### Regarding Test Interpretations

Incomplete test conduct occurred in the initial set-up stages of the study when participants were unable to submit their results through the app because of Wi-Fi connectivity issues. These were later resolved by resolving incompatibility issues of the app and the clinic’s Wi-Fi server.

Regarding test interpretations, a few participants mistakenly interpreted their negative result (the control line) as their positive result, despite instructions. An invalid result was also recorded that was truly negative.

Regarding test results, 3 participants tested positive for HIV (0.7% seropositivity) with both the self-test and rapid test, which were rapidly confirmed by laboratory results, and the participants were linked to a physician within the same day (linkage: 3/3, 100%) and returned for a follow-up appointment. All negative rapid and self-test results (448/451, 99.3%) were confirmed negative through laboratory testing, and all were linked to counseling (448/448, 100%). Lab testing conformed to the national testing algorithms.

Regarding linkages, all participants (451/451, 100%) were linked to in-person counseling following the self-testing procedure. All participants used the phone line and later met the research nurse. The average turnaround time to linkage to counseling ranged from 2 to 6 hours.

Regarding the preference for counseling, participants were in favor of counseling over the phone, followed by face-to-face counseling in a clinic. Some favored counseling over the internet (chat or website; 132/421, 31.4%) or in a pharmacy (121/421, 28.7%), both followed by face-to-face counseling in clinic and counseling in clinics only (132/421, 31.4%). Participants were generally not in favor of no face-to-face counseling (28/421, 6.7%) or no counseling at all (3/421, 0.7%).

Regarding cost preferences, half of the participants (206/421, 48.8%) selected Can $10-20 and 27.4% (115/421) selected <Can $10. In terms of the usefulness of the app, 98.8% (417/422) of the participants found the app helpful in guiding them through the self-testing process. Finally, 94.3% (395/419) of the participants said that they would recommend this self-testing strategy to their partner.

**Table 2:** Participant information collected through the HIVSmart! survey (n=438).

Information		Value, n (%)
**Gender (self-identified)**		
	Male	432 (98.6)
	Transgender	2 (0.5)
	I do not wish to answer	3 (0.7)
	Other	1 (0.2)
**Sexual orientation**		
	Homosexual	400 (91.3)
	Bisexual	32 (7.3)
	Heterosexual	4 (0.9)
	I do not wish to answer	1 (0.2)
	Other	1 (0.2)
**Highest level of education**		
	High school degree not completed	13 (3.0)
	High school	51 (11.6)
	College or technical school	125 (28.5)
	Undergraduate degree	169 (38.6)
	Graduate degree (master’s or PhD)	77 (17.6)
	Other	1 (0.2)
	I do not wish to answer	2 (0.5)
**Employment status**		
	Unemployed	46 (10.5)
	Employed	348 (79.5)
	Other	36 (8.2)
	I do not wish to answer	8 (1.8)
**Tested for HIV in the past 6 months**		
	No, I’ve never been tested	25 (5.7)
	Yes, in the last 6 months	230 (52.5)
	Yes, more than 6 months ago	178 (40.6)
	I have been tested, but did not want to receive my result	1 (0.2)
	I do not know if I have ever been tested	3 (0.7)
	I do not wish to answer	1 (0.2)
**Sexually active**		
	No	16 (3.7)
	Yes	421 (96.1)
	I do not wish to answer	1 (0.2)
**Number of different sexual partners in the past 6 months**		
	0	20 (4.6)
	1	38 (8.7)
	2-5	174 (39.7)
	6-10	110 (25.1)
	≥11	96 (21.9)
**In the past 6 months:**		
	Had sex without a condom	307 (70.1)
	Had sex with an HIV-infected partner	74 (16.9)
	Had sex with a sex worker	18 (4.1)
	Had sex under the influence of alcohol	195 (44.5)
	Had sex under the influence of drugs	83 (18.9)
	Had sex with multiple partners	219 (50.5)
	Injected drugs (excluding medicine)	5 (1.1)
**Exposure to HIV (eg, needles) in the workplace in the past 6 months**		
	No	426 (97.3)
	Yes	10 (2.3)
	I do not wish to answer	2 (0.5)

## Discussion

### Principal Findings

In this Canadian Institutes of Health Research funded innovative Canadian study, we investigated the feasibility of implementing an app-optimized unsupervised HIV self-testing strategy in a clinical setting.

Our unsupervised self-testing strategy was found to be feasible to operationalize (419/451, 96.6%), was well accepted (451/458, 98.5%), and preferred by participants. HIV was detected, and all participants were linked to care within a working day. All of our self-testers were linked to counseling or directed to a physician within hours, an essential service to offer with self-testing. Participants found the app-based approach to be a useful (417/422, 98.8%) in completing the HIV self-testing procedure, and a majority (395/419, 94.3%) wanted to recommend it to their friend.

This app-optimized, self-testing strategy was aimed to plug gaps in the self-testing process that are associated with an accurate detection, self-test interpretation, and rapid initiation of linkages to counseling and care. We were limited by the lack of approved HIV self-tests in Canada, which restricted our evaluation to a clinic environment [19-21] instead of homes. To stimulate a home environment, we set up kiosks in clinics where participants could test unsupervised, yet a nurse was always available to offer counseling and support [22]. Regarding costs, we found that a majority of participants believed that an acceptable price to pay for an HIV self-test in Canada was between Can $10 and $20. This finding is in line with 3 studies from New York City, where participants felt that an affordable and accessible price point was US $15-$25 (compared with the prevailing US $40) [23-25].

This is the first Canadian study to report data on the use of an app-based strategy. Many digital innovations (ie, Web-based programs, kiosk-based tablets, and short message service [SMS] text messaging services) are available, yet a complete, portable, and patient-friendly app-based solution for self-testing from engagement to linkage to counseling and care is novel [26].

In 5 studies that have evaluated some digital innovations, we observed a few limitations in their offer of services that impacted the process. A Dutch study evaluated a Web-based strategy, where participants could purchase self-tests and access self-test instructions and counseling, but they did not provide data or information on linkages [27]. Of 4 US studies, 2 reported positive findings on the feasibility of use of a kiosk tablet for HIV self-testing in emergency rooms but reported poor engagement (50%) of participants and limited data on detection and linkages [28,29]. Another US study evaluating the use of SMS text messaging self-test results only by participants found that it was preferred by participants [30]. The fourth study provided written and Web-based video instructions to participants to choose a picture that resembled their self-test result and reported comparable results to ours, with 100% of positive OraQuick results and 98% of negative results being interpreted correctly [31]. None of these studies evaluated linkages to care.

The HIVSmart! app is an integrated innovation. It offers a personalized experience of self-testing from access to linkage, which is a step up from a website-, tablet-, or SMS text messaging-only service and is housed in a secure Health Insurance Portability and Accountability Act-compliant cloud-based platform. Integrated innovations [32], like HIVSmart!, are a new trend in the digital innovations space, as reported in a recent systematic review [33]. In 2011-2013, we evaluated a Web-based HIVSmart! strategy successfully in South African health care professionals [17]. We are currently testing the strategy at scale in South African townships in a project funded by both the Governments of South Africa and Canada [18]. A prototype of this strategy was evaluated in students way back in 2009 [12].

With the increasing availability of PrEP in Canada, and the desire to self-test frequently expressed by those on PrEP, we included a subsample of participants on PrEP in this study. However, neither our main outcomes nor HIV status differed by the PrEP status. Our study was underpowered to detect subgroup differences (small number of PrEP participants). Studies suggest that PrEP increases the window period for seroconversion, taking longer to get a positive test result [34,35]. Yet, many of our self-test results were consistent with the rapid test results. Concordant with a study in PrEP users from Kenya, participants were in favor of self-testing [36].

### Limitation

A limitation of this study included convenience sampling that raises a concern of selection bias.

### Conclusions

In Canada, future research with HIV self-tests from provinces with high rates of undocumented HIV infection (Saskatchewan) and marginalized populations with undiagnosed HIV infection is warranted. Future research that incorporates digital strategies to plug service delivery gaps important for HIV self-testing will make it easier to offer and document self-testing.

Rapid approvals by Food and Drug Administration, Conformité Européenne, and World Health Organization Prequalification of self-tests, both oral and blood-based HIV self-tests, will help expand options to self-test in Canada. It will also increase the visibility of HIV self-tests in pharmacies, clinics, and outreach settings and democratize the process of HIV self-testing. Finally, the adoption of proven digital solutions will help improve engagement and expedite rapid linkages to care. Doing so will help address the last mile problem of detecting undiagnosed HIV infection in marginalized Canadians, thereby accelerating progress toward Joint United Nations Programme on HIV/AIDS 90-90-90 targets in Canada.

We conclude that the HIVSmart! app-optimized strategy is feasible, accepted, and preferred by an educated, urban MSM population of Montreal. With the app, participants were able to perform, interpret, store results, and rapidly link to care. The HIVSmart!-optimized, self-testing strategy could be adapted and contextualized to many at-risk populations within Canada and worldwide, thereby maximizing its public health impact.

## Abbreviations

ITA: Investigational Testing Authorization
MSM: men who have sex with men
PEP: postexposure prophylaxis
PrEP: pre-exposure prophylaxis
SMS: short service message
